# Trading Off Global Fuel Supply, CO_2_ Emissions and Sustainable Development

**DOI:** 10.1371/journal.pone.0149406

**Published:** 2016-03-09

**Authors:** Liam Wagner, Ian Ross, John Foster, Ben Hankamer

**Affiliations:** 1 Economics, Griffith Business School, Griffith University, Brisbane, Queensland, Australia; 2 IMB, The University of Queensland, Brisbane, Queensland, Australia; 3 School of Economics, The University of Queensland, Brisbane, Queensland, Australia; University of Vermont, UNITED STATES

## Abstract

The United Nations Conference on Climate Change (Paris 2015) reached an international agreement to keep the rise in global average temperature ‘well below 2°C’ and to ‘aim to limit the increase to 1.5°C’. These reductions will have to be made in the face of rising global energy demand. Here a thoroughly validated dynamic econometric model (Eq 1) is used to forecast global energy demand growth (International Energy Agency and BP), which is driven by an increase of the global population (UN), energy use per person and real GDP (World Bank and Maddison). Even relatively conservative assumptions put a severe upward pressure on forecast global energy demand and highlight three areas of concern. First, is the potential for an exponential increase of fossil fuel consumption, if renewable energy systems are not rapidly scaled up. Second, implementation of internationally mandated CO_2_ emission controls are forecast to place serious constraints on fossil fuel use from ~2030 onward, raising energy security implications. Third is the challenge of maintaining the international ‘pro-growth’ strategy being used to meet poverty alleviation targets, while reducing CO_2_ emissions. Our findings place global economists and environmentalists on the same side as they indicate that the scale up of CO_2_ neutral renewable energy systems is not only important to protect against climate change, but to enhance global energy security by reducing our dependence of fossil fuels and to provide a sustainable basis for economic development and poverty alleviation. Very hard choices will have to be made to achieve ‘sustainable development’ goals.

## Introduction

**Fig 1:**
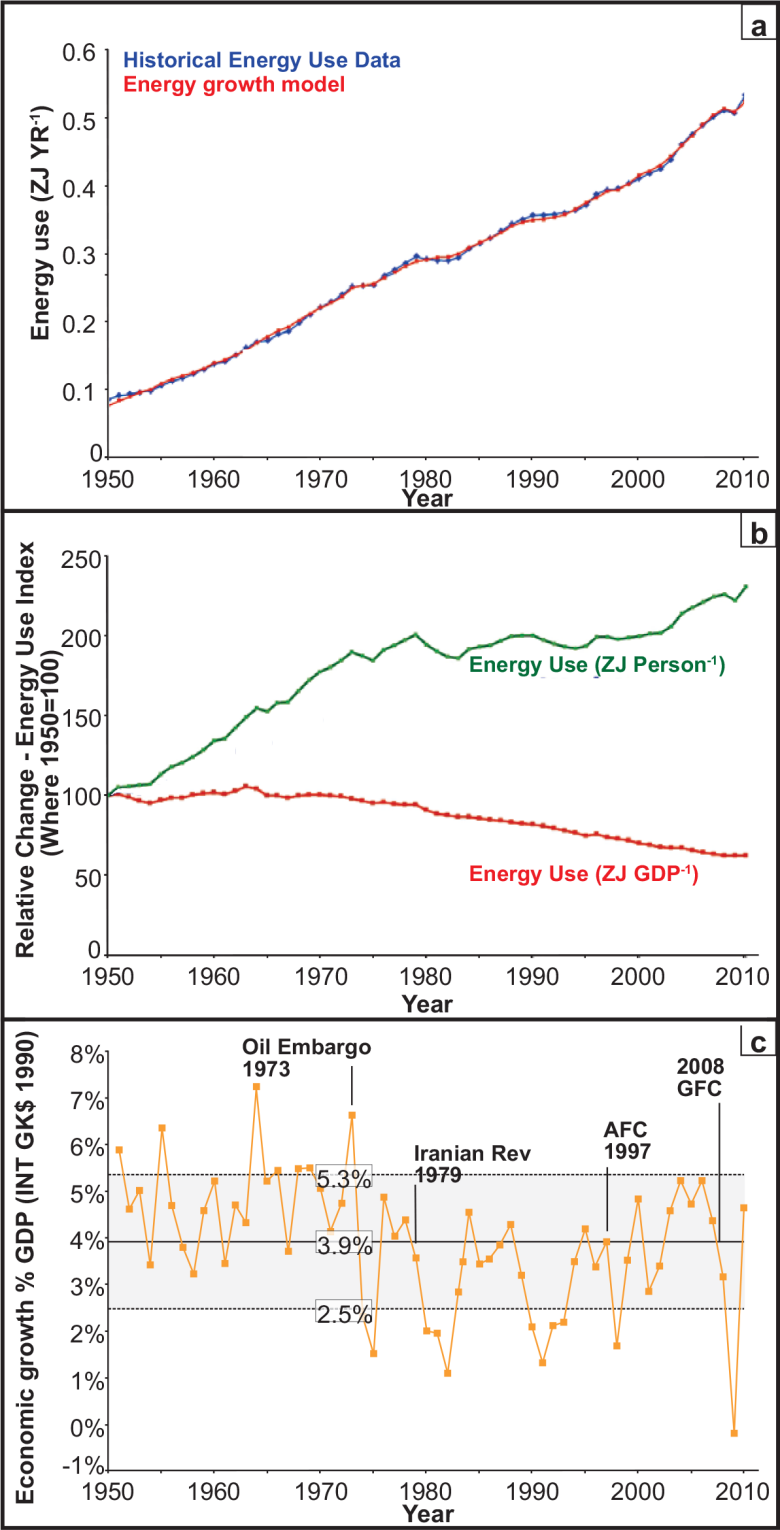
Energy use model: (A) Energy growth predictions compared with historical energy use data. (B) Historical energy use change in ZJ person^-1^ (Individual energy use) and ZJ GDP^-1^ (Economy-wide energy use). (C) Historical economic growth rates.

**Fig 2:**
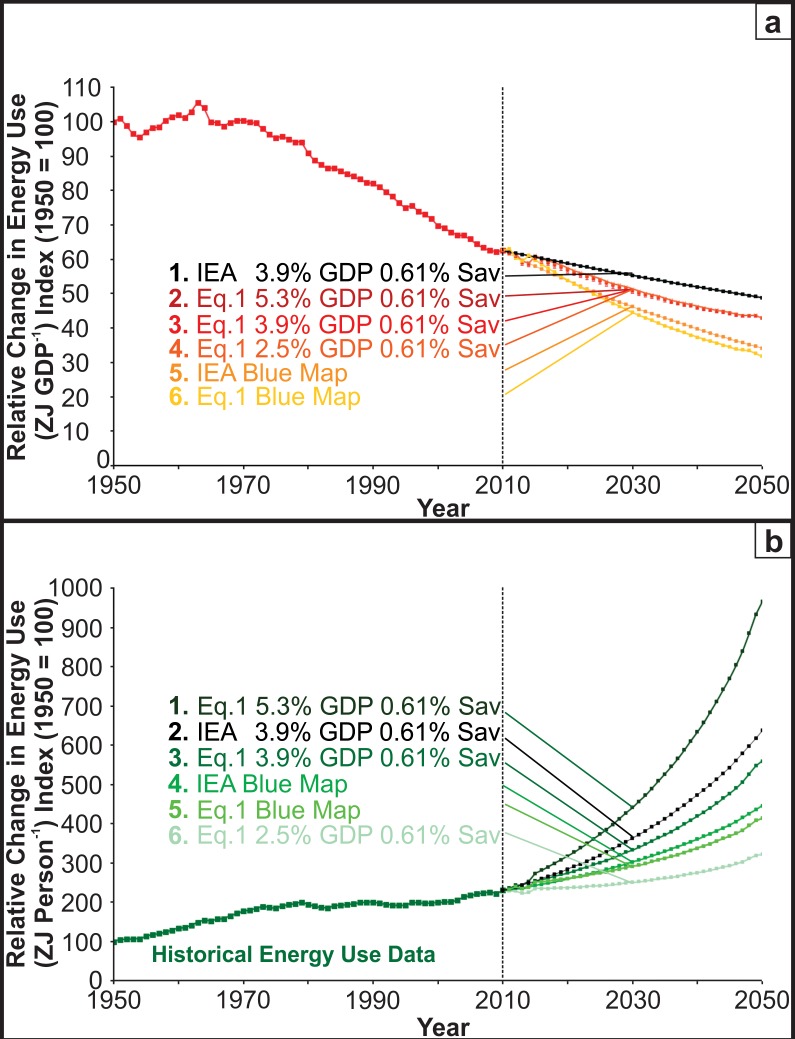
Historical data and forecasts (to the right of the dotted lines) are shown for relative change in energy use in ZJ GDP^-1^ (A), and ZJ person^-1^ (B) for a range of scenarios.

The global economy is valued at ~$100tn pa [1] and is powered by the $6tn energy sector [2]. By 2050, expansion of the human population to more than 9 billion people and continued global economic growth (3.9% pa growth since 1950) [1], will necessitate 50% more fuel [3] and CO_2_ emissions cuts of 80% [4], to maintain political, social, fuel and climate security. In this context extensive studies have been conducted on the documentation of coal [5,6], gas [7], oil [8–10], nuclear [11] and renewable energy sources [3,12] as well as historical [13] and forecast use [3,12] of these energy sources at the national [14–17] and the international level [15,18–22]. The effect of energy security on economics [23,24] and population growth on energy demand [25] have also been reported. This paper builds on this strong literature base by presenting a powerful validated global energy use tracker (Fig 1) which accurately accounts for this data and provides significant advantages over existing models [3,12,26]. The model is based on the three key variables: Population, energy use per person and economic activity (gross domestic product, GDP). Fig 1 and extensive statistical testing (see S1 File), strongly suggest that these variables are both ‘necessary and sufficient’ to track global energy demand over the past 60 years. It provides a solid basis for examining fuel demand with respect to changing global economic and population driven conditions over a similar time frame. The model uses a single standard common denominator energy unit (Joules) to replace the plethora of other units (e.g. million barrels of oil equivalent (Mbbl), British thermal units (BTU), thousand cubic feet of gas (TCF) and for electricity kilowatt hours (kWh)). This allows technology substitution to be accounted for based on the cost advantages of a given technology over time as well as improvements in the conversion process. The model is fully described and has been proven through rigorous testing using robust and freely available data and practices (see S1 File). It has also been validated against prominent IEA and EIA reports [3,12,26]. Importantly it extends beyond the IEA (Blue map target [3]) and EIA models by enabling the critical analysis of all major interacting factors (i.e. population, GDP, energy use person^-1^ and energy use GDP^-1^) the effects of which appear to have been underestimated in the IEA and EIA reports (Fig 2). In contrast our model shows that a dominant factor driving global energy demand is not energy use GDP^-1^, but energy use person^-1^ which is forecast to rise rises rapidly towards 2050, while the efficiency of production/conversion only gradually improves. The model’s ability to account for these interactions provides international policy makers with new tools and insights to guide the development of improved global energy security models and to assist with the development of effective emissions reductions and poverty alleviation scenarios. Importantly these capabilities challenge the common assumption of the EIA and IEA that increasing efficiency (energy use GDP^-1^) will solve our future energy supply problems.

## Methodology

This modelling uses Maddison’s GDP data set [27] which is the only annual data set for global GDP extending back to 1950. The model is estimated using least-squares regression and yields an R^2^ value of 0.84 which is remarkably high for a model specified with a dependent variable that is a rate of growth. It passed all required statistical and econometric tests indicating a high level of reliability for forecasting exercises (See Methods and S1 File). The proportional change in energy use over time (*d*ln(Energy(t)) is dependent on the contemporaneous proportional change in GDP (*d*ln(GDP(t)) and the proportional change in population (*d*lnPop(t-2) and *d*lnPop(t-4)) plus the (log) levels of energy use (ln(Energy(t-1)), population (lnPop(t-1)) and GDP (lnGDP(t-1)). The coefficients of Eq 1 are: α = 1.143692; β = -1.992702; χ = 4.559912; ε = -0.134103; φ = 0.129659; ρ = -0.066769. The chosen base year for energy use is 1950 (0.085 ZJ)[13].

Historical GDP (1990 International Geary–Khamis dollars) and GDP growth (%) for 1950 to 2008 were obtained from the Maddison data set [27]. The GDP and GDP person^-1^ values for 2009 and 2010 were taken from a World Bank data set [1] and suitably adjusted to be consistent with the Maddison data up to 2010 (See S1 File). This estimation has since been confirmed [28]. Historical world population was obtained from the same World Bank data set [1] and assumed to stabilize at 9 Billion from 2047, in line with UN medium population growth scenario [29]. Global energy use since 1950 was sourced from [13] and updated and cross checked with the IEA [12].

Documented 1P resources consist of 8.4 ZJ of oil [8,12,30], 6.66 ZJ of natural gas [12,30,31], 20.65 ZJ of coal [5,12,30] and 0.787 ZJ of Uranium [11]). URR consist of 22.77 ZJ of oil [9,10], 28.42 ZJ of natural gas [12], 30 ZJ of coal [5] and 1.57 ZJ of Uranium [11]).

The pathways for the growth in energy use were plotted to show non-renewable fuel depletion trajectories at varying rates of GDP (3.9% ± 1.4% yr^-1^ since 1950). To impose the additional constraint on changes in energy use per unit GDP (ZJ GDP^-1^) with the implementation of the proposed IEA Blue Map target, an upper limit on the amount of energy used as economic growth occurs was imposed.

The Breusch-Godfrey Serial Correlation Lagrange multiplier test [32,33], (see **Table B in S1 File**), was performed to demonstrate that the model does not suffer from any serial dependence between data points. The Durbin–Watson statistic [34,35] (see **Table B in S1 File**) additionally shows that this model does not suffer from serial correlation given the time lag components of the model. These tests which are fully described in the S1 File, demonstrate that this dynamic model does not suffer from serial correlation effects. Furthermore, to test for stationarity properties of the time series used in this study we have used the Augmented Dickey-Fuller (ADF) test [36,37]. As shown in **Table B** to **G in S1 File**, the null and alternative hypothesis can be rejected given the t-statistic values being smaller than the required critical values prescribed by Mackinnon [38,39].

S1 File provides detailed statistical analyses of our estimated model via tests for Serial Correlation, Heteroscedasticity, Ramsey RESET, Unit Roots and Co-Integration (**Table A** through to **Figure A in S1 File** and **Table H in S1 File** in the Supporting Information). These tests show that the explanatory variables of the model are both necessary and sufficient to describe the growth of energy use.

Using data from 1950 to 2010, the observed relationship between global energy demand (Fig 1A), global gross domestic product and global population was modelled. This model was specified in growth rates (annual first differences of the natural logarithms of variables) plus the natural logarithms of the levels of variables. The model is dynamic and allows for lags in impact. A ‘general-to-specific' methodology was used to yield a parsimonious model through the elimination of statistically insignificant lags. The structure of this model is reported in Eq 1 with the lags denoted in parenthesis:

dLn(Energy(t))=α⋅dln(GDP(t))+β⋅dln(Pop(t−2))+χ⋅dln(Pop(t−4))+ε⋅ln(Energy(t−1)+φ⋅ln(GDP(t−1))+ρln(Pop(t−1))) (1)

The estimated coefficients obtained are: α = 1.14 (11.48); β = -1.99 (-2.30); χ = 4.559912 (5.07); ε = -0.134103 (-4.36); φ = 0.129659 (3.64); ρ = -0.066769 (-3.33) with t-values reported in parenthesis. The chosen base year for global energy use is 1950 (0.085 ZJ)[13]. The length of the estimation period is constrained by the Maddison’s global GDP data set [27] which is the only collection to provide annual data that goes as far back as 1950. The model passes a stringent battery of statistical and econometric tests (see S1 File).

The model captures the relationship between global energy demand, global GDP and global population over recent history. A tight correlation is observed (Fig 1). There is a large literature on the direction of causation between energy and GDP which is inconclusive and this is unsurprising given their intimate connection [40]. Here we make no assumption concerning the direction of causality since it is not required for the forecasting exercises undertaken. Our aim was to establish for a given GDP growth rate, what the correlated energy demand growth is forecasted to be, irrespective of direction of causation. Under different conditions either energy availability (e.g. 1973) or GDP growth (e.g. 2008) might drive causality; the model simply draws the correlation curves and the user can decide whether the input values of the correlated variables are realistic or not. Widely accepted global projections for population growth have been used, and GDP per capita rates are based on historical ranges [27,28]. Forecasting scenarios are presented for different assumptions concerning global population growth and economic growth. Future risks to energy security and CO_2_ emissions targets were then evaluated (Fig 2).

The independent variables specified as rates of change capture short term impacts and those in levels capture long term impacts. To summarise, in the short term, a 1% increase in GDP growth is associated with a 1.14% increase in energy demand growth. The long term impact of a yearly 1% increase in population *growth* is associated with a net 4.56% yr^-1^ increase in the growth of energy demand. Population growth increases the demand for energy sharply in the short term; however this is moderated by falling energy use per capita (See Fig 1A and Fig 1B).

It should be noted that the estimated coefficients ε and φ are similar in size but have opposite signs. When we restrict them to be equal by entering *(lnEnergy(t-1)–lnGDP(t-1))* as a single independent variable, the reported result is very similar to an estimated coefficient of -0.14. What this suggests is that, as energy has been used more efficiently over time, energy demand has grown in response. This is evidence in support of the hypothesis that the Jevons paradox, or ‘rebound’ effect has been in operation [41].

### Historical energy use

International Energy Agency modelling of energy demand assumes that energy use is highly correlated with the raw measure of economic activity (GDP) [12]. At the global level it is therefore often assumed that, over time, energy efficiency improvements in production contributing to GDP, will broadly be achieved in most economic sectors and that this will enable global energy demand to be controlled. Indeed energy use per unit of GDP (ZJ GDP^-1^) has decreased by 37% (0.61% yr^-1^ energy efficiency) since 1950 (Fig 1B red). This improvement in energy efficiency was largely achieved through increasing knowledge and innovation which has driven technological energy efficiency [42]. However in accordance with the above Jevons paradox example, rather than reducing energy consumption per person (ZJ Person^-1^), individuals globally have used 2.17% yr^-1^ more energy (Fig 1B green) or 130% more since 1950. The historical energy use data (Fig 1B) shows that improvements in energy efficiency, raise growth in energy use. It is also the case that energy demand increases significantly faster than population in the short term because the damping effect of energy efficiency operates over a longer time lag. Thus an increase in energy efficiency associated with GDP (Fig 1B red) is also offset by population growth effects which are associated with the second part of the Jevons paradox. It should be emphasised that this additional energy use is not "discretionary" in the usual sense of the word. It reflects the fact that a higher standard of living intrinsically requires more energy (via underlying production, regulation and standards, redundancy and range of services). While some energy is "wasted" by consumers, most of this additional consumption is due to structural changes that cannot be removed without a discernible downgrading of quality of life (e.g. poverty alleviation).

In summary, although production efficiency per unit of *GDP* level has increased, each person uses more energy at the same time as global population rises. Thus, the potential for rapidly increasing energy demand in the future is high as global population is conservatively estimated to increase towards the widely predicted 9 billion by 2050 and possibly beyond [42].

The result reported in Eq 1 moves beyond the findings of generally accepted models estimated by the International Energy Agency and the Energy Information Agency (DOE). This is because the latter are based on energy use per unit of production (ZJ GDP^-1^; Fig 1B red) but do not reflect global patterns of energy use per person (ZJ Person^-1^; Fig 1B green). As most governments promote economic growth, poverty alleviation and increased energy equality, using only ZJ GDP^-1^ is inadequate. Eq 1 accounts for both simultaneously (see also Fig 1A).

### A transition in energy use

Fig 1A charts actual and predicted global energy use time series data from 1950–2010. The approximately linear trend was likely due to a relatively stable fraction of the global population (i.e. the G20 nations) using the bulk of global energy (83% in 2010). The rest of the world used a much smaller fraction. From 2000 to 2010 however, the economies of China (10.38% yr^-1^ since 2000; compounded GDP increase since 2000 = 196%) and India (7.47% yr^-1^ since 2000; compounded GDP increase since 2000 = 120%) in particular, expanded rapidly. Fig 1A clearly shows that the trend has begun to steepen since 2000.

### Global economic growth

Given the importance of global GDP as a variable in Eq 1, its historical range was examined (Fig 1C: 3.9% +/-1.4% GDP yr^-1^). Fig 1C also shows that GDP growth is quite volatile. Large short-term increases in energy prices, as in 1973 and 1979, reduce planned production with consequent falls in GDP and the consumption of energy. This short run effect is confirmed in Eq 1 and results in an estimated coefficient on GDP growth in excess of unity, indicating energy use is highly influenced by changes in economic conditions when all other variables remain constant. GDP growth fell rapidly from 4.57% in 1973 to 0.41% in 1975 and similarly during the Iranian revolution (1979). After the OPEC oil embargo of 1973, oil prices quadrupled to $96 bbl^-1^ in 2009 USD while the Iranian Revolution saw Dubai light oil prices [30] rise from ~$13 bbl^-1^ to ~$30 bbl^-1^.

The drop in GDP in both of these examples was equivalent to ~75% of the decline in GDP during the recent Global Financial Crisis. This illustrates a crucial mechanism that is likely to be operative into the future: restrictions in energy supply, whether induced by a cartel or rising costs, raise energy prices and this in turn leads to reductions in production and, thus, GDP and lower energy consumption. The opposite may occur for a limited period as oil prices drop due to oversupply as was the case in 2015. However, the operation of the model is agnostic about which variable is the "driver".

### Energy efficiency

The global economic growth rate (3.9%±1.4%yr^-1^ since 1950) [27] has only dropped below 2% during major recessions. This growth rate profile resulted in an average annual increase in energy use of 2.17% person^-1^ (Fig 1B green). In comparison, global energy efficiency savings have been significantly lower (0.61%yr^-1^). This is consistent with the IEA technology perspectives report which concluded that the OECD 11 countries have achieved annual energy efficiency improvements of 0.7% since 1973 [3]. To improve upon this, the International Energy Agency has proposed a ‘Blue Map Target’ with an additional 0.8% yr^-1^ energy savings from 2011, yielding 1.41% savings in total [3]. But the rate of increase in energy use (2.17% person^-1^ yr^-1^) remains significantly higher than any of the above energy saving scenarios (0%, 0.61% or 1.41%). So, if the world continues along a business-as-usual track (3.9% economic growth yr^-1^ globally; 0.61% energy savings yr^-1^) a rapid increase in global energy demand is expected, even if the global population remained constant. Conversely, a growth in energy demand which matches either observed or aspirational energy efficiency gains, necessarily requires significantly lower GDP growth than historical averages. We conclude that energy demand will most likely continue to rise even in the face of modest GDP per capita growth. The forecast global population increase from ~7 billion in 2008 to ~9 billion people by 2050 and possibly to 14 billion by 2100, compounds this problem and will likely have serious implications for economic, energy and climate change policy even if the additional population lives in poverty and uses little energy.

### Energy demand forecasting

Diagnostic testing of the econometric global energy demand model (Eq 1; Fig 1A) suggests that it is a robust forecasting tool for the exploration of different scenarios. Fig 2A (red) and Fig 2B (green) are colour coded to match Fig 1B; red (ZJ GDP^-1^) and green (ZJ Population^-1^). In each case the data to the left of the dotted lines (Fig 2A and 2B), represent the data shown in Fig 1B but in relative change units. To the right of the dotted line a range of forecasts for different scenarios are shown.

### Economic energy use (ZJ GDP^-1^)

Economic energy use refers to energy use per unit of GDP. The baseline model from the IEA (Fig 2A curve 1) shows the relative change in ZJ GDP^-1^ at the historical GDP growth rate (3.9%) and energy savings (0.61%). It forecasts energy efficiency improvements of 24.5% between 2010–2050. The corresponding Eq 1 model (Fig 2A curve 3) forecasts a similar energy efficiency improvement (38% for 2010–2050). The difference between these two models is accounted for by the fact that our model is based on a longer time series (IEA from 1973; Eq 1 from 1950).

The IEA Blue map target (Fig 2A curve 5) represents a 0.8% energy savings per year on top of the historical savings rate of 0.61% from 1950–2010 (total energy savings = 1.41% per year). It forecasts energy efficiency improvements of 60% between 2010 and 2050. The corresponding Eq 1 Blue map implementation (Fig 2A curve 6), also adds an energy efficiency improvement rate of 0.8% to the endogenised historical energy efficiency rate of 0.61% at the historical GDP average of 3.9% (total energy saving = 1.41% per year). It forecasts a similar energy efficiency improvement (69% between 2010–2050). This confirms close agreement between Eq 1 and IEA modelling in terms of ZJ GDP^-1^ energy use, both for the baseline models (Fig 2A curves 1 & 3) and the IEA and Eq 1 Blue map models (Fig 2A curves 5 & 6).

As expected, varying the economic growth rate (Fig 2A curves 4 (2.5%), 3 (3.9%) and 2 (5.3%) yr^-1^ GDP) did not significantly alter energy efficiency improvements between 2010–2050 (37.5%, 38% and 38.2% respectively) in terms of ZJ GDP^-1^, further validating the model in Eq 1.

### Individual energy use (ZJ population^-1^)

Fig 2B shows that individual energy use (ZJ population^-1^) is strongly influenced by GDP. Fig 2B (curve 6) illustrates the lowest GDP level (2.5% yr^-1^) and forecasts the lowest rate of change in energy use (-92% between 2010–2050, i.e. -2.3% yr^-1^). This is followed by 3.9% GDP growth models with the highest energy savings rates (Fig 2B (curves 5 and 4; IEA and Eq 1 Blue map targets). These models forecast increases in energy efficiency of -184% (-4.6% yr^-1^) and -216% (-5.4% yr^-1^) over the 2010–2050 period, respectively. The 3.9% IEA and Eq 1 GDP growth models with the historical 0.61% yr^-1^ energy savings rate (Fig 2B curves 3 and 2) resulted in higher energy use and forecast increases in energy efficiency of -230% (-5.75% yr^-1^) and -408% (-10.2% yr^-1^) over the 2010–2050 period, respectively. The greatest increase in energy use was for the 5.3% GDP growth rate scenario (Fig 2B curve 1). This forecasts an increase in energy efficiency of -737% over the 2010–2050 period (-18.42% yr^-1^). This marked effect of GDP on individual energy use (ZJ person^-1^) is likely due to increased production efficiency (Fig 1B red ZJ GDP^-1^) resulting in reduced product prices, or a perceived increase in personal wealth. This results in a higher individual energy demand rate (Fig 2B). So rather than stabilizing our energy use through increased production efficiency (Fig 2A) the dominant factor affecting global energy use appears to be energy use per person (Fig 2B). Indeed continuing along a business as usual track (Fig 2B curve 2) is forecast to result in a ~300% increase in global energy demand by 2050. This is at first surprising. However ~50% of the global population has an income of ~$2.50 per day, and so the aspirational goal of most policy makers, is to remain on a continuous economic growth track to increase prosperity and enable poverty alleviation. However this is forecast to result in a very rapid increase in energy demand.

In summary, Eq 1 provides an improved method for examining different forecasting scenarios for energy use, based on ZJ GDP^-1^, and ZJ Person^-1^ [12,26]. This provides a basis for estimating fossil fuel depletion rates, based on reported reserves [43].

### Forecasting Fossil Fuel Depletion

**Fig 3:**
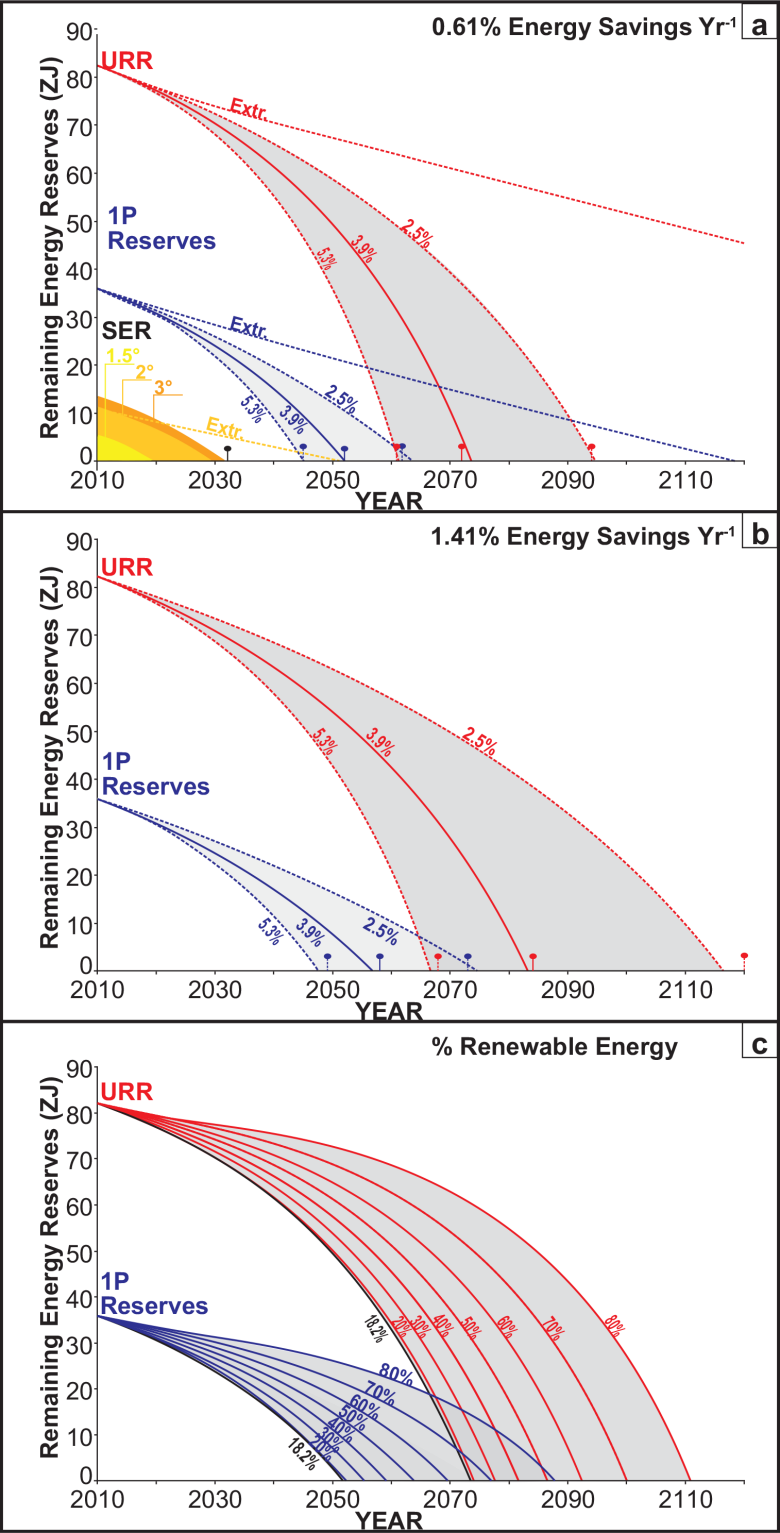
Fossil fuel depletion model: (A) Modelling of the depletion of Safely Extractable Reserves (SER) to meet 1.5 (yellow: 480GtC), 2(mid yellow: 570GtC) and 3°C (orange: 609GtC) global warming targets proposed in [44–46], 1P reserves (red) and URRs (blue) at the historical energy savings rate of 0.61% yr-1 and (B) the 1.41% yr-1 Blue map target using Eq 1. Fuel depletion trajectories are shown at economic growth rates of 2.5%, 3.5% and 5.9% based on the +/- 1 SD historical rate range (Fig 1C). ‘Extr’ extrapolates the 1950–2010 energy use rate. The pin markers indicate the corresponding depletion dates based on IEA methodology. (C) Models the effect of increasing renewable energy contribution from the current 18.2% level up to 80% in URRs (red), IP reserves (blue).

Reported total fossil fuel reserves vary considerably (36 to 712 ZJ [5,7,8,12]); this influences depletion dates calculated from them and provides latitude for both optimists, who assume that prices will expand reserves greatly and pessimists who regard cheaper and more efficient extraction techniques as simply hastening the inevitable depletion. The higher estimates account for all predicted reserves, including those that are likely to be too expensive or technically challenging to extract. For the purposes of this paper, more conservative ZJ values were also calculated based on the literature [5,8,12,30] via a weighted average for tonnage and the quality of coal, gas and oil incorporated in each fuel type class (see S1 File). 1P reserves (90% probability of recovery) and Ultimately Recoverable Resources (URR—5% probability of recovery) at current fuel prices were determined to be 36.5ZJ (1P) and 82.7ZJ (URR) respectively (see methods) [5,11,12,30]. Fuel reserves were converted to a common ZJ value to allow total global fossil fuel depletion rates to be forecast (Fig 3) based on realistic settings for economic growth and population using Eq 1. For the Fig 3 forecasts, population was assumed to rise from its current level of ~7 to 9 billion by 2050 and then to stabilize in accordance with the UN’s medium population growth scenario [29]. The economic growth rates were set at 3.9% ±1.4% yr^-1^ to represent a range of one standard deviation (SD) since 1950 (Fig 1C 2.5%, 3.9% and 5.3%). In Fig 2A the historical energy savings rate of 0.61% yr^-1^ was applied. As the global economy is highly dependent on fossil fuels (82% of global energy supply), this ‘business-as-usual’ model assumes that the percentage of fossil fuel use will remain constant and that, within this pool, fossil fuels will be used in an interchangeable manner. This approach was taken to demonstrate, via a set of the fossil fuel depletion trajectories, that without access to additional energy sources increasingly severe supply constraints are predicted even for the larger reserve sizes. This is considered to be a conservative estimate, given the rapid rise of global energy demand (Fig 2B: Eq 1 3.9% GDP scenario, curve 3) and the current global economic climate. It is uncertain whether the rate of renewable energy system deployment will be sufficiently fast to maintain a renewable energy market share of approximately 18.2%.

### 1P and URR

A key conclusion from Fig 3A, is that, with an international ‘pro-growth’ focus in most economies and a goal of alleviating poverty internationally (both of which require the maintenance of historical GDP growth rates), fossil fuel depletion is not forecast to proceed in a linear manner. The rapid rise in global population, the industrialization of developing nations coupled with compounding economic growth, are the primary factors that can transform growth of energy use from a relatively linear path (Fig 1A) to one which more closely resembles exponential growth (Fig 2B). This contrasts with the linear trend observed between 1950–2000 (accurately modelled *log-linearly* in Eq 1) which is likely due to a relatively small fraction of the global population (i.e. G20 nations) using the bulk of global energy (83% in 2010). Currently 50% of the global population lives on less than $2.50 per day (low energy demand).

Setting population to 6.845 billion in Eq 1 has the effect of extrapolating the historical linear trend observed between 1950–2010 into the future (Fig 3A Extr.) and results in fossil fuel depletion dates of (URR:2271) and IP (2120). However this requires historically low GDP per capita growth, and effectively excludes an additional 31% of the 2050 global population from the benefits of economic growth. This is inconsistent with international ‘pro-growth’ strategies aimed at alleviating poverty and assumes that poorer nations will be content to (or be compelled to) remain that way.

Fig 3A forecasts energy depletion for a business-as-usual scenario (2.5%–5.3% economic growth–see Fig 1C; 0.61% Energy savings per year) for the whole global population, in which 1P and URRs would be depleted much earlier (1P Reserves between 2047–2065 (blue curves); URRs between 2063–2096 (red curves)). The blue and red ‘pin’ markers indicate independent forecasts based on IEA methodology which strongly support Eq 1 forecasts. Both models indicate that all documented 1P reserves would be depleted within ~50 years and all reported URR (many of which are classified as only having a 5% chance of recovery) within ~80 years, if GDP growth rates tracks within 3.9% ± 1 SD range observed since 1950 (Fig 1C).

It has long been argued that that estimates of URR are deeply uncertain due to the difficulties associated with prospecting and extraction, because energy prospecting is driven by demand. In this view, rising energy prices will greatly expand reserves. However, it has been shown that, as EROI falls, price rises nonlinearly with respect to supply [47,48]. Given the documented on-going fall in EROI of fossil fuels over the last two decades, and the fact that recent increases in supply come from better extraction technologies rather than new reserves, it is unlikely that rising prices will expand supply sufficiently to meet the exponential increase in demand that would be produced by global GDP growth in a business-as-usual model. Furthermore EROI values of greater than 3 are reported to be required to extract sufficient energy to enable the infrastructure of modern economies to be maintained [49].

### Safely Extractable Reserves (SER)

Using a weighted average (56Mt-CO_2_/EJ) the combustion of the 1P resources (36 ZJ) would result in the release of ~2044 billion tonnes of CO_2_. This is >3.4 times greater than the 600 billion tonne limit that can be combusted if we remain within the 2°C global warming ‘safe limit’ of the Intergovernmental Panel on Climate Change imposed at the United Nations Conference on Climate Change (Paris 2015). Following a business-as-usual scenario for the total global population (3.9% GDP, 0.61% energy efficiency savings) would result in Safely extractable reserves within the 2°C limit being depletion by ~2029 (Fig 3A light orange) [45]. This suggests that by 2030 the CO_2_ emissions from the global economy should minimally be in balance with the sustainable rate of global CO_2_ absorption [50,51] (i.e. ~ 48% of global anthropogenic CO_2_ emissions in 2010 [50,51], if the 2°C global warming ‘safe limit’ of the Intergovernmental Panel on Climate Change is not to be exceeded [4,52]. To stay within a 1.5°C global warming limit, safely extractable reserves are forecast to be consumed by 2020 (Fig 3A yellow). While it is possible that these time-points can be shifted back through rapid adoption of renewables, the degree to which this is possible is severely limited by the short time frame available to do so. Even the 3°C limit will, according to this model will be very challenging to meet by 2033 (Fig 3A dark orange)

This model is supported by the fact that Global CO_2_ emissions are tracking at the upper levels forecast by the IPCC [53,54] as well as by the US DOE which forecasts that energy use will rise to 0.721–0.852 ZJ by 2035 with economic growth rates ranging from 2.5% (Low), 3.9% (Medium) and 5.3% (High), scenarios. In comparison, our models yield 2035 energy use values of 0.759ZJ (2.5% GDP), 1.08 ZJ (3.9% GDP), 1.528 ZJ (5.3% GDP). While at low GDP growth rates our model agrees well with that of the EIA, it diverges significantly as GDP rises. This is likely due to the fact the EIA model [26], does not take ZJ person^-1^ into account, which is strongly affected by GDP. ‘Extr’ extrapolates the 1950–2010 energy use rate (Fig 3A light orang dotted line). The pin markers indicate the corresponding depletion dates based on IEA methodology. Fig 3(C) models the effect of increasing renewable energy contribution from the current 18.2% level up to 80% in URRs (red), IP reserves (blue).

### Energy efficiency and renewable energy

To evaluate the effect of increasing energy efficiency, fuel depletion scenarios based on the Eq 1 Blue map trajectories (1.41% yr^-1^) were also modelled (Fig 3B). This 1.41% yr^-1^ energy savings rate is 2.31 times higher than the historical average of 0.61% since 1950. Even energy savings measures of this magnitude only extend 1P reserve forecasts (Fig 3B blue curves) by approximately a decade (2049–2075) and URRs by ~20 years (2068–2117). Similarly, increasing the percentage of renewable energy supply from the current 18.2% to an 80% renewables level is forecast to extend 1P reserves from 2052–2088 (Fig 3C blue curves) and URRs to 2074–2112 (Fig 3C red curves) illustrating the forecast increase in energy demand per person (Fig 2B).

## Discussion

Energy is essential to human survival and underpins all economy-wide (Fig 1C&1D) and individual (Fig 2B) activities. Using global population and GDP data, as specified in Eq 1, it is possible to model the growth of global energy use robustly, over the 1950–2010 period (Fig 1A). This provides a solid basis for forecasting energy use (Fig 2A and 2B), fossil fuel depletion (Fig 3) and future CO_2_ emissions under different scenarios more reliably than IEA models.

Historical data clearly show that energy security is essential to economic, social and political stability (Fig 1C OPEC Oil Embargo and the Iranian revolution). Our modelling supports a forecast of continued rise in energy demand which, if supplied mainly by fossil fuels, would result in fuel supply constraints by mid-century. Meanwhile, internationally stated commitments are to alleviate poverty through ‘pro-growth’ strategies and to simultaneously stay well below the IPCC 2°C ‘safe limit’ by reducing CO_2_ emissions [44,46].

Our modelling argues that it is not possible to attain all three goals with fossil fuels alone. Even maintaining GDP growth per capita at historical levels will lead to energy supply constraints within a few decades, with the sharpest price rises towards the end of this time, due to projected population growth. To innovate away from fossil fuel dependence (~80% of demand in 2010) requires considerable time, as low-emissions fuel capacity is difficult to expand rapidly regardless of price increases.

Furthermore, recent reports suggest that the safe limit should be lowered to a ~1.5°C temperature rise, further restricting Safely Extractable Reserves (Fig 3A) [45,55]. This position was advocated by 106 of the 195 countries who attended the United Nations Conference on Climate Change (Paris 2015). This group of nations (e.g. Alliance of Small Island States (AOSIS), the Climate Vulnerable Forum) represents over 1 billion people most vulnerable to climate change.

### Securing supply

Because CO_2_ as a ‘negative externality’ has not been priced into production in every jurisdiction the continued level of subsidies by national governments has led to a global market failure and earlier onset of climate change [56]. Furthermore, the worst consequences of unprecedentedly high greenhouse gas levels on the atmosphere are decades away, and are therefore heavily discounted by current economic analysis [56]. Thus, without government intervention we have arrived at the situation where the inadequately regulated free market may not be capable of effecting a rapid enough transition to sustainable long term CO_2_ neutral energy systems.

Most of the global population is at the lower end of the income range with few effective ways to shift away from fossil fuel consumption quickly (e.g. via the installation of solar panels, micro-hydro and wind generators), except through the use of readily available biomass (e.g. via deforestation, which would likely result in extensive environmental damage). When governments try to intervene to provide a workable set of incentives to reduce carbon emissions, fossil fuel industries have demonstrated strong resistance to efforts to control greenhouse gases and the price of carbon [57].

Based on results of IEA modelling [3,12] to date, the advent of CO_2_ sequestration technology designed to allow continued use of fossil fuels seems less likely than the possibility of alternative low-C energy sources making up the shortfall. This is because the same price signals that could drive expansion of fossil fuel use if coupled to CO_2_ sequestration, also assist the viability of other low-C technologies. It is quite clear from the results presented here, that even if the expected entry timing of utility scale CCS technology is met [3,12], this will still result in a failure to adequately reduce CO_2_ levels and our results question the ability for this technology to maintain a reliable energy supply in light of the Safely Extractable Reserve constraint (see Fig 3A).

The results reported in this paper suggest that even stabilizing fossil fuel use will be politically challenging. Despite the >1000% increase in non-hydro renewables between 1990 and 2014 renewable energy systems deployment, the percentage of energy derived from renewables has not increased at a rate capable of keeping up with the growth in global energy demand and only makes a small contribution to primary energy supplies. To achieve significant CO_2_ emissions reductions without a requires:

1. the prolonged reduction of global economic growth to levels lower than those prevailing after the recent Global Financial Crisis (which negatively impacts poverty alleviation)
2. a reduction in population growth more rapid than generally projected for example through increased equality, education and employment of women (reduction not yet noted)
3. a significantly increased energy efficiency (e.g. the Blue map target) beyond historical precedent and/or
4. a rapid transition to CO_2_ neutral renewable energy sources.

Based on this we conclude that globally it is essential to accelerate the transition to sustainable long term, CO_2_-neutral energy systems if continued prosperity is to be achieved. Tapping into the huge energy resource of the sun (3020 ZJ yr^-1^ vs. ~0.56ZJ yr^-1^ total global energy demand) is one such option both to produce electricity (20% of global energy demand) and fuels (80% of energy demand) [58].

Our findings show that it is critically important that policy makers factor in the potentially rapid decline not only of 1P and URR’s as well as the limits posed by ‘Safely Extractable Reserves (SER). It is particularly important to establish whether it is economically advantageous to continue investing heavily in next generation fossil fuel-based infrastructure for relatively short term gain, rather than transitioning in a controlled but rapid manner to renewable energy technologies that are capable of supporting the global economy into the future. Markets may flexibly and efficiently meet the need for sustainable energy systems, but only if global governments set the required legal frameworks.

### Potential transition strategy

The question of the relative costs of fossil fuels and sustainable low-emissions energy systems can be partially addressed by examining current subsidies. A transition to long term CO_2_ neutral energy systems could be supported through the global reduction of oil and coal industry subsidies, with cost-savings facilitating the establishment of new low-C-emissions fuel industries. Clearly, expediting the introduction of effective and workable international carbon tax/trading schemes to encourage CO_2_ neutral technology deployment is also desirable. The International Monetary Fund has estimated that the global cost of subsidizing gasoline, diesel and kerosene exceeded US$500 billion per year in 2008. Furthermore, the IEA in 2012 estimated that the consumer subsidy for all fossil fuels to be US$523 billion [12,59]. [60]. The costs of climate change are higher and have been estimated ~ 1% per year of GDP [56,61], ~US$755 Billion (PPP 2014) [1]. The removal of fossil fuel subsidies and the phase out of nuclear power in conjunction with the implementation climate change mitigation strategies is forecast to result in an only small decrease in GDP (-0.3% in 2035 [59]) which is well within the standard deviation observed over the last 60 years. The annual fossil fuel subsidy is, the equivalent of ~US$18bbl [60] and corresponds to approximately 10% of the ~$6tn global energy sector [2]. Importantly these subsidies have the effect of locking in the use of fossil fuel based energy sources and slowing down the uptake of clean energy alternatives. Governments could, in a cost neutral manner, correct the prevailing subsidies and incentives in a way that would protect against fuel poverty while encouraging fuel security, CO_2_ emissions reductions and sustainable long term economic stability. This could for example be achieved by settings increasingly stringent EROI and greenhouse gas emissions targets over time and transitioning subsidies from current fossil fuel technologies to those technologies capable of meeting them.

### Environment and economy

At the Paris Climate Change Summit, firm CO_2_ reduction targets were implemented to restrain global temperatures rises to ≤2°C. However, the perception that this target will have negative impacts on national economies, as with the previous Copenhagen and Cancun Climate Change Summits persists. In contrast, our findings strongly suggest that persistently seeking high economic growth through fossil fuel use will not only accelerate CO_2_ emissions but eventually induce a fuel security problem which could have a catastrophic effect on many poor people in developing countries facing higher energy prices, as well as leading to increased consequences of climate change. Our scenarios place global economists and environmentalists on the same side as reductions in CO_2_ emissions and the enhancement of energy security (and, thus, human economic welfare) both require significant reductions of fossil fuel combustion. Whether or not the global private sector can foresee or address the exhaustion of reserves and enact rapid switches to alternative sources of energy remains an open question. The noted tendency for businesses to heavily discount the future in making investment decisions would suggest that such a transition will be slow unless appropriate price incentives are put in place to compensate for ‘market failures’. This seems to be the primary role of governments but they are constrained by short term political considerations that make long term environmental policy very difficult to enact, unless bipartisan support can be secured for defined and enforceable targets. We note, however, that the sheer scale of sustainable fuel supply required makes this a massive, long-term stable global market which promises significant financial gain for successful companies.

## Supporting Information

S1 File PLOS_supps_v3.docx. General Statistical Tests for the Energy Growth Model. (Table A). Multi-Variable Linear Model Represented as a Difference Equation of the Change in Levels, with Residuals (Figure A). Breusch-Godfrey Serial Correlation LM Test (Table B). Heteroskedasticity Test: Breusch-Pagan-Godfrey (Table C). Ramsey RESET Test (Table D). Augmented Dickey-Fuller Unit Root Test on DLGDP (Table E). Augmented Dickey-Fuller Unit Root Test on DLENERGY (Table F). Augmented Dickey-Fuller Unit Root Test on DLPOP (Table G). Johansen Co-Integration Test Summary (Table H).
(DOCX)
