# Supplementary material for: Trading Off Global Fuel Supply, CO2 Emissions and Sustainable Development
Source: PLoS One. 2016 Mar 9;11(3):e0149406. doi: 10.1371/journal.pone.0149406 (PMC4784947; doi:10.1371/journal.pone.0149406)
Supplement: S1 File — General Statistical Tests for the Energy Growth Model. (Table A). Multi-Variable Linear Model Represented as a Difference Equation of the Change in Levels, with Residuals (Figure A). Breusch-Godfrey Serial Correlation LM Test (Table B). Heteroskedasticity Test: Breusch-Pagan-Godfrey (Table C). Ramsey RESET Test (Table D). Augmented Dickey-Fuller Unit Root Test on DLGDP (Table E). Augmented Dickey-Fuller Unit Root Test on DLENERGY (Table F). Augmented Dickey-Fuller Unit Root Test on DLPOP (Table G). Johansen Co-Integration Test Summary (Table H). (DOCX) [file pone.0149406.s001.docx]

**SUPPORTING INFORMATION**

Historical GDP (1990 International Geary–Khamis dollars) and GDP growth (%) for 1950 to 2008 were obtained from the Maddison data set (1). The GDP and GDP person^-1^ for 2009 and 2010 were estimated using World Bank (2), by establishing the change in Log(GDP) and then applying this change in levels to the Maddison data set for 2009 and 2010. This estimation has since been confirmed by (3). Historical World population was obtained from the World Bank (2) and assumed to stabilize at 9 Billion from 2047 in line with the UN medium population growth scenario (4). Global energy use since 1950 was sourced from (5) and updated and cross checked with the IEA (6).

Following data compilation, a model of first differences of natural logarithms was established using the change in levels of the GDP, GDP person^-1^ and population over the time horizon of 1950-2010. This equation can be interpreted as an ‘equilibrium correction’ model. (Eq.1)

|  | $\begin{matrix} DLn(Energy(t))=\alpha D\ln(GDP(t))+\beta D\ln(Pop(t-2))+\chi D\ln(Pop(t-4)) \\ +\varepsilon\ln(GDP(t-1)+\phi\ln(Pop(t-1)+\rho\ln(Energy(t-1)) \end{matrix}$. | (Eq. 1) |
| --- | --- | --- |

and calibrated with the method of least squares using the statistical package EViews (7) with the following coefficients: α=1.143692; β=-1.992702; χ=4.559912; ε=-0.134103; φ=0.129659; ρ=-0.066769. The statistical significance of the model is verified via its R^2^=0.84. The supplementary materials below provide detailed analysis of our model via tests for Serial Correlation, Heteroskedasticity, Ramsey RESET, Unit Roots and Co-Integration (Supporting.Tables.1-8 and Supporting .Fig.1 below). These tests show that the model's variables are necessary and sufficient to describe the growth of energy use. Using the historical data (GDP, GDP person^-1^ and energy use), the model shows its closeness of fit to the actual energy consumption data (See Fig.1.a) as well as the change in energy use with respect to GDP (ZJ GDP^-1^, Fig.1d), GDP person^-1^ (ZJ GDP^-1^ person^-1^, Fig.1e) and Population (ZJ Person^-1^, Fig.1f).

Documented 1P resources consist of 8.4 ZJ of oil (6, 8, 9), 6.66 ZJ of natural gas (6, 9, 10), 20.65 ZJ of coal (6, 9, 11) and 0.787 ZJ of Uranium (12)). URR consist of 22.77 ZJ of oil (13, 14), 28.42 ZJ of natural gas (6), 30 ZJ of coal (11) and 1.57 ZJ of Uranium (12)).

The pathways for the growth in energy use were plotted to show non-renewable fuel depletion trajectories at varying rates of GDP (3.9% ± 1.4% yr^-1^ since 1950). Further, to impose the additional constraint on changes in energy use per unit GDP (ZJ GDP^-1^) with the implementation of the proposed IEA Blue Map target simply imposes an upper limit on the amount of energy used as economic growth occurs.

**References:**

1. Maddison A (2007) *The world economy volume 1: A millennial perspective volume 2: Historical statistics* (Academic Foundation).

2. World Bank (2011) World Development Indicators. (World Bank).

3. Bolt J & van Zanden JL (2013) The First Update of the Maddison Project; Re-Estimating Growth Before 1820. *Maddison-Project Working Paper WP-4, University of Groningen, January* 5.

4. UN Department of Economic & Social Affairs Population Division (2004) World Population to 2300. ed Secretariat TDoEaSAotUN.

5. Grubler A (1998) *Technology and Global Change* (Cambridge University Press).

6. IEA (2012) *World Energy Outlook, 2012* (International Energy Agency).

7. Software' QM (2007) *EViews 6 Command Reference.* (Irvine CA, USA.).

8. Mohr SH & Evans GM (2010) Long term prediction of unconventional oil production. *Energ Policy* 38(1):265-276.

9. BP (2013) Statistical Review of World Energy 2013. (London).

10. ENI (2013) World Oil and Gas Review.

11. Mohr SH, Evans, G. M. (2009) Forecasting coal production until 2100. *Fuel* 88(11):2059-2067

12. OECD Nuclear Energy Agency (2007) *Uranium 2007: Resources, Production and Demand* (OECD).

13. Bentley RW, S. A. Mannan and S. J. Wheeler. (2007) Assessing the date of the global oil peak: The need to use 2P reserves. *Energ Policy* 35:6364-6382.

14. Kjarstad J, Johnsson. F. (2009) Resources and future supply of oil. *Energ Policy* 37:441-464.

**Table A: General Statistical Tests for the Energy Growth Model.**

| Dependent Variable: DLEnergy  Method: Least Squares  Sample (Adjusted): 1955-2010  Included observations: 56 after adjustments | | | | |
| --- | --- | --- | --- | --- |
|  | Coefficient | Std. Error | t-Statistic | Prob. |
| DLGDP | 1.143692 | 0.099668 | 11.47505 | 0.0000 |
| DLPOP(-2) | -1.992702 | 0.866639 | -2.299346 | 0.0257 |
| DLPOP(-4) | 4.559912 | 0.899423 | 5.069817 | 0.0000 |
| LENERGY(-1) | -0.134103 | 0.030753 | -4.360596 | 0.0001 |
| LGDP(-1) | 0.129659 | 0.035576 | 3.644600 | 0.0006 |
| LPOP(-1) | -0.066769 | 0.020029 | -3.333670 | 0.0016 |
| R-squared | 0.840744 | Mean dependent var | | 0.030232 |
| Adjusted R-Squared | 0.824819 | S.D. dependent var | | 0.021624 |
| S.E. of Regression | 0.009051 | Akaike info criterion | | -6.471011 |
| Sum squared residuals | 0.004096 | Schwarz criterion | | -6.254009 |
| Log Likelihood | 187.1883 | Hannan-Quinn crierion | | -6.386879 |
| Durbin-Watson Stat | 2.111300 |  | |  |


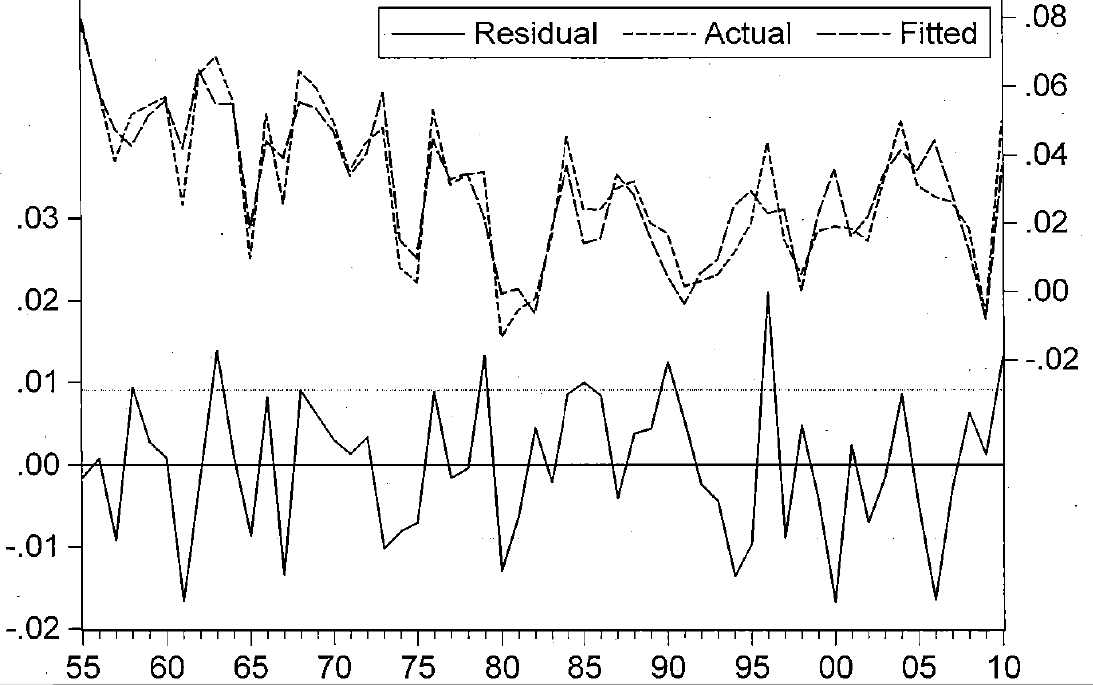


Figure A: Multi-Variable Linear Model Represented as a Difference Equation of the Change in Levels, with Residuals

**Table B: Breusch-Godfrey Serial Correlation LM Test**

| F-Statistic | 0.677999 | | | Prob. F(2,48) | | 0.5124 | |
| --- | --- | --- | --- | --- | --- | --- | --- |
| Obs*R-squared | 1.538534 | | | Prob. Chi-Squared(2) | | 0.4634 | |
| Test Equation:  Dependent Variable: RESID  Method: Least Squares  Sample: 1955-2010  Included observations: 56  Pre-sample missing value lagged residuals set to zero | | | | | | | |
|  | | Coefficient | Std. Error | | t-Statistic | | Prob. |
| DLGDP | | 0.001781 | 0.100329 | | 0.017752 | | 0.9859 |
| DLPOP(-2) | | 0.215079 | 0.892336 | | 0.241029 | | 0.8106 |
| DLPOP(-4) | | -0.233591 | 0.927894 | | -0.251743 | | 0.8023 |
| LENERGY(-1) | | 0.007293 | 0.031725 | | 0.229875 | | 0.8192 |
| LGDP(-1) | | -0.007386 | 0.036525 | | -0.202216 | | 0.8406 |
| LPOP(-1) | | 0.003755 | 0.20501 | | 0.183151 | | 0.8555 |
| RESID(-1) | | -0.110136 | 0.152134 | | -0.723940 | | 0.4726 |
| RESID(-2) | | -0.155636 | 0.153345 | | -1.014940 | | 0.3152 |
| R-squared | | 0.027474 | Mean dependent var | | | | 7.61E-07 |
| Adjusted R-Squared | | -0.114353 | S.D. dependent var | | | | 0.008629 |
| S.E of Regression | | 0.009109 | Akaike info criterion | | | | -6.427440 |
| Sum squared residuals | | 0.003983 | Schwarz criterion | | | | -6.138104 |
| Log Likelihood | | 187.9683 | Hannan-Quinn crierion | | | | -6.315265 |
| Durbin-Watson Stat | | 1.948903 |  | | | |  |

**Table C: Heteroskedasticity Test: Breusch-Pagan-Godfrey**

| F-Statistic | 0.498350 | | | Prob. F(6,49) | | 0.8065 | |
| --- | --- | --- | --- | --- | --- | --- | --- |
| Obs*R-squared | 3.220723 | | | Prob. Chi-Squared(6) | | 0.7807 | |
| Scaled explained SS | 1.927469 | | | Prob. Chi-Squared(6) | | 0.9262 | |
| Test Equation:  Dependent Variable: RESID^2  Method: Least Squares  Sample: 1955-2010  Included observations: 56 | | | | | | | |
|  | | Coefficient | Std. Error | | t-Statistic | | Prob. |
| C | | -0.004583 | 0.009462 | | -0.484397 | | 0.6303 |
| DLGDP | | 0.000521 | 0.00103 | | 0.505570 | | 0.6154 |
| DLPOP(-2) | | -0.011525 | 0.009214 | | -1.250823 | | 0.2169 |
| DLPOP(-4) | | 0.003896 | 0.009333 | | 0.417453 | | 0.6782 |
| LENERGY(-1) | | 0.000178 | 0.000317 | | 0.562173 | | 0.5766 |
| LGDP(-1) | | -0.00345 | 0.000446 | | -0.773770 | | 0.4428 |
| LPOP(-1) | | 0.000430 | 0.000655 | | 0.656482 | | 0.5146 |
| R-squared | | 0.057513 | Mean dependent var | | | | 7.31E-05 |
| Adjusted R-Squared | | -0.057894 | S.D. dependent var | | | | 9.04E-05 |
| S.E. of Regression | | 9.30E-05 | Akaike info criterion | | | | -15.61130 |
| Sum squared residuals | | 4.24E-07 | Schwarz criterion | | | | -15.35813 |
| Log Likelihood | | 444.1163 | Hannan-Quinn crierion | | | | -15.51314 |
| F-Statistic | | 0.498350 | Durbin-Watson Stat | | | | 2.293550 |
| Prob (F-Statistic) | | 0.806490 |  | | | |  |

**Table D: Ramsey RESET Test**

| F-Statistic | | 0.207717 | | Prob. F(6,49) | | 0.6506 | |
| --- | --- | --- | --- | --- | --- | --- | --- |
| Log Likelihood ratio | | 0.236889 | | Prob. Chi-Squared(6) | | 0.6265 | |
| Test Equation:  Dependent Variable: DLENERGY  Method: Least Squares  Sample: 1955-2010  Included observations: 56 | | | | | | | |
|  | Coefficient | | Std. Error | | t-Statistic | | Prob. |
| DLGDP | 1.065797 | | 0.198253 | | 5.375932 | | 0.0000 |
| DLPOP(-2) | -1.792865 | | 0.977451 | | -1.834225 | | 0.0727 |
| DLPOP(-4) | 4.192812 | | 1.212752 | | 3.457270 | | 0.0011 |
| LENERGY(-1) | -0.120800 | | 0.042579 | | -2.837076 | | 0.0066 |
| LGDP(-1) | 0.117780 | | 0.044333 | | 2.656684 | | 0.0106 |
| LPOP(-1) | -0.060961 | | 0.023874 | | -2.553433 | | 0.0138 |
| Fitted^2 | 1.388625 | | 3.046836 | | 0.455760 | | 0.6506 |
| R-squared | 0.841416 | | Mean dependent var | | | | 0.030232 |
| Adjusted R-Squared | 0.821998 | | S.D. dependent var | | | | 0.021624 |
| S.E. of Regression | 0.009123 | | Akaike info criterion | | | | -6.439527 |
| Sum squared residuals | 0.004078 | | Schwarz criterion | | | | -6.186358 |
| Log Likelihood | 187.3067 | | Hannan-Quinn crierion | | | | -6.341373 |
| Durbin-Watson Stat | 2.110961 | |  | | | |  |

**Table E: Augmented Dickey-Fuller Unit Root Test on DLGDP**

| Null Hypothesis: DLGDP has a unit root  Exogenous: Constant  Lag Length: 0 (Automatic based on SIC, MAXLAG=10) | | | | | | | | |
| --- | --- | --- | --- | --- | --- | --- | --- | --- |
|  | | |  | | t-Statistic | | Prob* | |
| Augmented Dickey-Fuller test statistic | | | | | -5.274857 | | 0.0000 | |
| Test critical values: | | 1% level | | | -3.546099 | |  |  |
|  | | 5% level | | | -2.911730 | |  | |
|  | | 10% level | | | -2.593551 | |  | |
| *Mackinnon (1996) one sided p-values  Augmented Dickey-Fuller test Equation  Test Equation:  Dependent Variable: DLENERGY  Method: Least Squares  Sample: 1955-2010  Included observations: 56 with adjustments | | | | | | | | |
|  | Coefficient | | | Std. Error | | t-Statistic | | Prob. |
| DLGDP(-1) | -0.641879 | | | 0.121687 | | -5.274857 | | 0.0000 |
| C | 0.024336 | | | 0.004943 | | 4.923610 | | 0.0000 |
| R-squared | 0.328021 | | | Mean dependent var | | | | -0.000198 |
| Adjusted R-Squared | 0.316232 | | | S.D. dependent var | | | | 0.015538 |
| S.E. of Regression | 0.012848 | | | Akaike info criterion | | | | -5.837889 |
| Sum squared residuals | 0.009410 | | | Schwarz criterion | | | | -5.767464 |
| Log Likelihood | 174,2177 | | | Hannan-Quinn crierion | | | | -5.810397 |
| F-Statistic | 27.82412 | | | Durbin-Watson Stat | | | | 1.972287 |
| Prob (F-Statistic) | 0.000002 | | |  | | | |  |

**Table F: Augmented Dickey-Fuller Unit Root Test on DLENERGY**

| Null Hypothesis: DLENERGY has a unit root  Exogenous: Constant  Lag Length: 0 (Automatic based on SIC, MAXLAG=10) | | | | | | | | |
| --- | --- | --- | --- | --- | --- | --- | --- | --- |
|  | | |  | | t-Statistic | | Prob* | |
| Augmented Dickey-Fuller test statistic | | | | | -5.065583 | | 0.0001 | |
| Test critical values: | | 1% level | | | -3.546099 | |  |  |
|  | | 5% level | | | -2.911730 | |  | |
|  | | 10% level | | | -2.593551 | |  | |
| *Mackinnon (1996) one sided p-values  Augmented Dickey-Fuller test Equation  Test Equation:  Dependent Variable: D(DLENERGY)  Method: Least Squares  Sample: 1952-2010  Included observations: 59 with adjustments | | | | | | | | |
|  | Coefficient | | | Std. Error | | t-Statistic | | Prob. |
| DLENERGY(-1) | -5.99889 | | | 0.118425 | | -5.065583 | | 0.0000 |
| C | 0.017805 | | | 0.004382 | | 4.063143 | | 0.0001 |
| R-squared | 0.310429 | | | Mean dependent var | | | | -0.000317 |
| Adjusted R-Squared | 0.298332 | | | S.D. dependent var | | | | 0.023204 |
| S.E. of Regression | 0.019437 | | | Akaike info criterion | | | | -5.009939 |
| Sum squared residuals | 0.021535 | | | Schwarz criterion | | | | -4.939514 |
| Log Likelihood | 149.7932 | | | Hannan-Quinn crierion | | | | -4.982448 |
| F-Statistic | 25.66013 | | | Durbin-Watson Stat | | | | 1.900962 |
| Prob (F-Statistic) | 0.000005 | | |  | | | |  |

**Table G: Augmented Dickey-Fuller Unit Root Test on DLPOP**

| Null Hypothesis: DLPOP has a unit root  Exogenous: Constant  Lag Length: 0 (Automatic based on SIC, MAXLAG=10) | | | | | | | | |
| --- | --- | --- | --- | --- | --- | --- | --- | --- |
|  | | |  | | t-Statistic | | Prob* | |
| Augmented Dickey-Fuller test statistic | | | | | -1.307259 | | 06207 | |
| Test critical values: | | 1% level | | | -3.546099 | |  |  |
|  | | 5% level | | | -2.911730 | |  | |
|  | | 10% level | | | -2.593551 | |  | |
| *Mackinnon (1996) one sided p-values  Augmented Dickey-Fuller test Equation  Test Equation:  Dependent Variable: D(DLPOP)  Method: Least Squares  Sample: 1952-2010  Included observations: 59 with adjustments | | | | | | | | |
|  | Coefficient | | | Std. Error | | t-Statistic | | Prob. |
| DLPOP(-1) | -0.074962 | | | 0.057343 | | -1.307259 | | 0.1964 |
| C | 0.001162 | | | 0.000971 | | 1.195636 | | 0.2368 |
| R-squared | 0.029108 | | | Mean dependent var | | | | -8.89E-05 |
| Adjusted R-Squared | 0.012075 | | | S.D. dependent var | | | | 0.001311 |
| S.E. of Regression | 0.001303 | | | Akaike info criterion | | | | -10.41542 |
| Sum squared residuals | 9.67E-05 | | | Schwarz criterion | | | | -10.34500 |
| Log Likelihood | 309.2550 | | | Hannan-Quinn crierion | | | | -10.38793 |
| F-Statistic | 1.708925 | | | Durbin-Watson Stat | | | | 1.986613 |
| Prob (F-Statistic) | 0.196374 | | |  | | | |  |

**Table H: Johansen Co-Integration Test Summary**

| Sample: 1950-2010  Included observations: 58  Series: LGDP LPOP LENERGY  Lags interval: 1 to 2  Selected (0.05 level*) Number of Co-Integration Relations by Model | | | | | |
| --- | --- | --- | --- | --- | --- |
| Data Trend: | None | None | Linear | Linear | Quadratic |
| Test Type | No Intercept | Intercept | Intercept | Intercept | Intercept |
|  | No Trend | No Trend | No Trend | Trend | Trend |
| Trace | 3 | 2 | 0 | 0 | 0 |
| Max-Eig | 0 | 0 | 0 | 0 | 0 |
| *Critical values based on Mackinnon-Haug-Michelis (1999)  Information Criteria by Rank and Model | | | | | |
| Data Trend: | None | None | Linear | Linear | Quadratic |
| Rank or | No Intercept | Intercept | Intercept | Intercept | Intercept |
| No. of CEs | No Trend | No Trend | No Trend | Trend | Trend |
|  | Log Likelihood by Rank (rows) and Model (columns) | | | | |
| 0 | 650.5516 | 650.5516 | 654.9760 | 654.9760 | 662.8854 |
| 1 | 658.4419 | 659.7107 | 664.1272 | 664.3228 | 669.7543 |
| 2 | 664.5447 | 666.2859 | 669.6543 | 670.5176 | 674.3580 |
| 3 | 668.0454 | 669.8673 | 669.8673 | 675.0961 | 675.0961 |
|  | Akaike Information Criteria by Rank (rows) and Model (columns) | | | | |
| 0 | -21.81212 | -21.81212 | -21.86124 | -21.86124 | -22.03053 |
| 1 | -21.87731 | -21.87731 | -21.96990 | -21.94216 | -22.06049* |
| 2 | -21.88085 | -21.87193 | -21.95360 | -21.91440 | -22.01234 |
| 3 | -21.79467 | -21.75404 | -21.75404 | -21.83090 | -21.83090 |
|  | Schwarz Criteria by Rank (rows) and Model (columns) | | | | |
| 0 | -21.17268 | -21.17268 | -21.11522 | -21.11522 | -21.17793 |
| 1 | -21.02471 | -20.99845 | -21.01073 | -20.94747 | -20.99475 |
| 2 | -20.81510 | -20.73513 | -20.78127 | -20.67103 | -20.73345 |
| 3 | -20.51577 | -20.36857 | -20.36857 | -20.33885 | -20.33885 |
